# Content validity of patient-reported outcomes for use in lower-risk myelodysplastic syndromes

**DOI:** 10.1186/s41687-020-00235-4

**Published:** 2020-08-26

**Authors:** Jeremiah J. Trudeau, Jianming He, Esther Rose, Charlotte Panter, Sharan Randhawa, Adam Gater

**Affiliations:** 1 Janssen Global Services LLC, 700 US 202 South, Raritan, NJ 08869 USA; 2 Janssen Pharmaceuticals LLC, Raritan, NJ 08869 USA; 3 Adelphi Values, Bollington, Cheshire UK

**Keywords:** Myelodysplastic syndrome, Patient reported outcomes, FACT-An, QUALMS, Health-related quality of life

## Abstract

**Background:**

The lower-risk (low and intermediate-1 risk based on IPSS) myelodysplastic syndrome (MDS) has a negative impact on patients’ health-related quality of life (HRQoL). Patient Reported Outcomes (PROs) instruments, which are used to collect patients’ HRQoL data, should have established content validity in the target population to ensure that the instrument is comprehensive and comprehensible. The present study was conducted to evaluate the content validity of the Quality of Life in Myelodysplasia Scale (QUALMS) and the Functional Assessment of Cancer Therapy-Anemia (FACT-An) PRO instruments in patients with lower-risk MDS.

**Methods:**

In this cross-sectional, qualitative study, 16 patients aged ≥18 years with lower-risk MDS, who were RBC transfusion dependent, literate and fluent in US-English were interviewed. Interviews were semi-structured comprising of two parts: concept elicitation (CE) explored symptoms and impacts important to patients, and cognitive debriefing (CD) assessed understanding and relevance of the QUALMS and FACT-An. A conceptual model was developed, which was used to map the concepts that emerged during CE onto the QUALMS and FACT-An to assess concept coverage and suitability of the instruments.

**Results:**

The median age of participants was 67.5 years (range: 51–91), with half being female (*n* = 8). Nine (56.2%) participants had intermediate-1-risk MDS and 10 (62.5%) were relapsed or refractory to erythropoiesis-stimulating agent treatment. Fatigue/tiredness (100.0%), shortness of breath (87.5%), weakness (81.2%), and low energy (75.0%) were reported most commonly and were the most bothersome symptoms as well. Of seven high-level HRQoL domains identified, activities of daily living (*n* = 16, 100.0%), physical functioning (*n* = 15, 93.8%), emotional wellbeing (*n* = 13, 81.3%), social functioning (*n* = 12, 75.0%), sleep disturbance (*n* = 9, 56.3%), and impact on work (*n* = 9, 56.3%) were the most commonly reported. For CD, the QUALMS and FACT-An were found to be mostly relevant and very well understood; response options were easy to use, and recall period was appropriate.

**Conclusion:**

Both QUALMS and FACT-An demonstrated a strong face and content validity in patients with lower-risk MDS, suggesting that these instruments are appropriate for assessing HRQoL in this population.

## Introduction

Myelodysplastic syndromes (MDS) are a heterogeneous group of clonal hematopoietic malignancies characterized by cytopenias and abnormal cell morphology [1]. Patients with MDS develop anemia, infections, bleeding, and are at a risk of progression to acute myeloid leukemia (AML), which is generally refractory to treatment, and associated with increased mortality [2]. Cytopenias are commonly associated with a high symptom burden including fatigue, weakness [3], shortness of breath, chest pain, and dizziness [4], resulting in a poor physical, functional, and social well-being [5], and an overall decreased health-related quality of life (HRQoL) compared with age and sex matched populations [6]. According to a 2015 estimate in the USA, prevalence of MDS may range between 60,000 to 170,000, and incidence among patients aged ≥65 years was estimated between 75 to 162 per 100,000 patients [7]. Burden of MDS may be underestimated and is projected to grow with improving life expectancy [8].

The International Prognostic Scoring System (IPSS) grouped patients’ condition into one of the two risk categories: lower-risk (IPSS: low and intermediate-1) or higher-risk (IPSS: intermediate-2 and high). Lower-risk MDS tends to progress slowly compared to the higher-risk MDS. A 2008 survey of physicians in the USA revealed that approximately 60–70% of newly diagnosed patients had lower-risk MDS [9], and a report from the European LeukaemiaNet MDS registry also agreed that most of the MDS patients are low or intermediate-1 risk [10]. With variable prognosis, a substantial proportion of lower-risk patients are still reported to have short survival [11]. The cause of death in patients with lower-risk MDS is mostly consequences of bone marrow failure, particularly infections, rather than progression to AML [12]. Lower-risk MDS is characterized by macrocytic anemia, which is treated with erythropoiesis-stimulating agents (ESAs), however the response duration is short [13–15]. Anemia with red blood cell (RBC) transfusion dependency and subsequent iron overload negatively impacts patients’ HRQoL and disease course [16–18]. Fatigue is one of the primary symptoms of anemia apart from lethargy, headache, dizziness, shortness of breath, pale skin, and heart palpitations [19, 20].

Patient-reported outcomes (PRO) data can explain the impact of a disease on patients’ HRQoL and support clinical decision making and regulatory claims for new therapies [21]. PRO data are an important complement to assess how patients feel and function when used in conjunction with other study endpoints like survival, transfusion independence or hematologic improvement [22]. In accordance with best practice guidelines for use of PRO instruments in clinical trials to support regulatory claims, content validity should be established in the target population to ensure that a PRO instrument assesses relevant concepts, is adequately comprehensive, and consistently interpreted as intended [23, 24].

The Quality of Life in Myelodysplasia Scale (QUALMS) is a 38-item MDS-specific PRO instrument that assesses HRQoL in patients with MDS [25]. The instrument comprises a 14-item physical burden subscale (QUALMS-P; e.g., item 8: *“...have you experienced shortness of breath?*”), 3-item benefit-finding subscale (QUALMS-BF; e.g., item 30: “*… how often did you feel you were able to find quality information about MDS treatments?”*) and 11-item emotional burden subscale (QUALMS-E; e.g., item 3: “*… how often did you feel as though you couldn’t do anything about your disease?”*). Additionally, there are five single-item questions presented at the end of the instrument not included in scoring, as they are not applicable to all patients. Higher QUALMS scores denote better MDS-related quality of life. The QUALMS was developed based on input from patients with MDS, their caregivers and clinicians [25–27]; and its validity, reliability, and responsiveness have been established in an MDS population [25]. However, evidence of the instruments’ content validity is limited; this is typically established based on qualitative research to provide evidence that the items and domains of an instrument are appropriate and comprehensive relative to its intended context of use, that being a lower-risk MDS population. Further, evidence of content validity must be established prior to evidence of construct validity, reliability or sensitivity to change being interpreted [28].

The Functional Assessment of Cancer Therapy-Anemia (FACT-An) is a 47-item cancer-specific PRO instrument commonly used to assess the effect of anemia on HRQoL of cancer patients [29]. The FACT-An consists of 27 general items as the core instrument (FACT-G) and an additional 20 anemia items (anemia subscale) [30]. The FACT-An consists of 13 fatigue items (e.g., item An2: *“I feel tired”*) and 7 non-fatigue items (e.g., item An10: *“I get headaches”*). The FACT-An, despite published evidence supporting its statistical reliability and validity in MDS populations, requires additional qualitative evidence of content validity in this context of use [29–32]. Therefore, the aim of the present study was to evaluate the content validity of the QUALMS and FACT-An questionnaires through qualitative concept elicitation (CE) and cognitive debriefing (CD) interviews with lower-risk MDS patients.

## Method

### Study design

This cross-sectional, non-interventional, qualitative study involved semi-structured interviews with 16 adults with lower-risk MDS in the US. A CE exercise was conducted to elicit spontaneous information about the patient experience of MDS. A CD exercise was also conducted to assess whether the QUALMS and FACT-An are well understood, relevant, and capture all concepts important to patients. The QUALMS and FACT-An collectively comprise 85 items (38 items and 47 items, respectively), which would have been difficult to fully debrief in a 90-min interview without undue burden on participants. Because the FACT-G has been used extensively in diverse cancer populations, only the FACT-An anemia subscale (20 items) was selected for debriefing of the FACT-An.

### Sample selection

Sample size determination in qualitative interview studies is based on the goal to achieve “concept saturation”, a point at which no new concepts are likely to emerge with further interviews [33, 34]. While it is difficult to project how many interviews are necessary to achieve saturation, experience suggests that 10–15 patients capture > 90% of concepts [35]. Therefore, it was anticipated that interviews with 16 MDS patients would be sufficient to obtain concept saturation and all relevant information, with additional interviews to be conducted if necessary.

Participants were recruited through clinician referrals from the Mayo Clinic, Rochester, US and patient advocacy groups – the MDS Foundation (https://www.mds-foundation.org/) and Rare Patient Voice. Eligible participants (male or female) were: aged ≥18 years with IPSS lower-risk MDS according to the WHO criteria classification [36], RBC transfusion dependent (requiring at least 2 transfusion events during an 8–12 week period), had an Eastern Cooperative Oncology Group (ECOG) performance status 0, 1 or 2; and literate and verbally fluent in US-English. ECOG score ranges from 0 to 5 (0: ‘fully active’; 1: restricted in physically strenuous activity, fully ambulatory and able to carry out light work; 2: ambulatory and capable of all selfcare but unable to carry out any work activities; 3: capable of only limited selfcare, confined to bed or chair more than 50% of waking hours; 4: completely disabled, cannot carry on any selfcare; 5: death) and used to assess patients’ general fitness and suitability for treatment, and to ensure homogeneity of a study sample with respect to the performance status [37]. Patients with history of hematopoietic stem cell transplant, those diagnosed with any malignancy other than MDS; and those with severe physical, neurological or cognitive condition that could interfere with participant’s ability to participate in a 90-min interview, hearing, speaking, reading, or understanding were excluded. A quota sampling approach (a non-probability sampling technique typically used in qualitative research) was used to ensure a representative sample selection.

### Ethics

The study was approved and monitored by an Independent Review Board (IRB) in the US (approval code: ADE1–17-061); local IRB approval was obtained at the Mayo clinic. Written informed consent was obtained prior to collection of any data and participants were paid for their involvement in the study. Participant data were stored, used, and disseminated according to the Health Insurance Portability and Accountability Act (HIPAA) legislation.

### Interview procedure

The interviews were 90-min each in length (comprising 20 min of CE and 70 min of CD) and conducted over telephone or video calling technology (Skype) by a trained interviewer using a semi-structured interview guide. All interviews were audio-recorded and transcribed verbatim for analysis, with all identifiable information redacted. The interview methodology employed in this study was in line with published guidance, which aims to ensure that qualitative research is rigorous and accurately captures the patient experience [23, 38, 39].

The CE exercise comprised of open-ended, exploratory questions designed to elicit spontaneous and unbiased responses about the patient experience with MDS. Subsequently, participants were probed with more focused questions on concepts that may not have been emerged during the open-ended interview. Participants were also directly asked which symptoms they experienced most often, and which symptoms were most bothersome. The findings were used to develop a conceptual model of MDS; specifically, the symptoms and impacts on functioning/HRQoL identified as the most important by participants were grouped into domains and displayed visually. The conceptual model was used to map the identified symptom and impact concepts onto both the QUALMS and entire FACT-An to assess concept coverage and suitability for assessing symptoms and impacts of MDS.

For the CD exercise, participants were asked to complete each questionnaire (the QUALMS and FACT-An anemia subscale) separately using a ‘think aloud’ approach [40], where they were asked to share their thoughts as they read each instruction and selected each response. This process helped to identify any aspects that were understood or interpreted incorrectly. Subsequently, participants were asked detailed questions about their interpretation and understanding of instructions and item wording, the relevance of concepts, the appropriateness of the response options and recall period, and whether any concepts were missing.

### Qualitative analysis

Transcripts were analyzed by sorting via thematic analysis methods using ATLAS.ti, a qualitative data analysis package (ATLAS.ti Scientific Software Development GmbH; Berlin, Germany). Participant quotes pertaining to the research questions were assigned concept codes in accordance with a provisional coding list that was agreed on with the research team after the first transcript was coded. The coding list was used throughout the coding/analysis process to ensure consistency and new codes were organically added throughout the process. Concepts elicited during the interview spontaneously or when probed by the interviewer were coded separately.

For the purposes of evaluating concept saturation, interview transcripts were chronologically grouped into three sets, two of these sets contained five transcripts and the third set contained six transcripts. Concepts emerging from the first two sets were compared, followed by comparison with the concepts that emerged from the third set. Saturation was deemed achieved if no new concept(s) emerged in the final set.

CD data were also coded using thematic analysis, and a frequency count for each item in the QUALMS and FACT-An anemia subscale was provided for understanding, relevance, appropriateness of response options, and adherence to, and appropriateness of recall period. Understanding was determined if participants were able to put the instruction/item into their own words. Understanding was deemed unclear if the participant provided a response to the item but did not expand on their understanding of the concept, or the participant simply repeated the item wording as written.

## Results

### Participant characteristics

Of the 16 participants included, half of the sample consisted of females (*n* = 8, 50.0%) and the median age was 67.5 years (range: 51–91 years). Majority of the participants (*n* = 9, 56.2%) had intermediate-1-risk MDS (Table 1**)**. In total, 10 participants (62.5%) had relapsed/ were refractory to ESA treatment, three (18.8%) were receiving ESA treatment, two (12.5%) had never received, and one (6.2%) was currently not receiving ESA treatment and provided no information regarding previous ESA treatment.

**Table 1:** Patients’ characteristics

Description	Total (*N* = 16)
**Demographic characteristics**	
Age, median (Range)	67.5 (51, 91)
Sex, n (%)	
Female	8 (50.0%)
Living status, n (%)	
Living with husband/wife/partner	12 (75.0%)
Living alone	3 (18.7%)
Living with parents/family or friends	1 (6.3%)
Work status, n (%)	
Retired	13 (81.3%)
Not working due to MDS	2 (12.5%)
Disability	1 (6.3%)
Race, n (%)	
Caucasian	15 (93.7%)
Black/African American	1 (6.3%)
Highest level of education, n (%)	
College or university	7 (43.8%)
Some years of college	5 (31.3%)
Graduation or professional degree	2 (12.5%)
Some high school	1 (6.3%)
High school diploma or general educational development	1 (6.3%)
**Clinical characteristics**	
Years since diagnosis, Median (Range)	3.6 (0.25, 50.8)
IPSS risk category, n (%)	
Intermediate-1-risk	9 (56.2)
Low-risk	7 (43.8)
MDS Subtype^a^, n (%)	
Refractory Anemia with Ring Sideroblasts (RARS)	7 (43.8)
Refractory Cytopenia with Multilineage Dysplasia (RCMD)	6 (37.5)
MDS Unclassified (MDS-U)	2 (12.5)
Refractory Cytopenia with Unilineage Dysplasia (RCUD)	1 (6.3)
MDS associated with del(5q) including the 5q- syndrome	1 (6.3)
ECOG performance status, n (%)	
0	7 (43.8)
1	9 (56.2)
Chronic diseases other than MDS, n (%)	8 (50.0)
Cardiovascular	6 (75.0)
Endocrinological/Nutritional	3 (37.5)
Rheumatological	2 (25.0)
Gastroenterological	1 (12.5)
Hematological	1 (12.5)
Gynecological	1 (12.5)
Dermatological	1 (12.5)
Current prescribed pharmacological treatment n (%)	
Erythropoietin	3 (18.8)
Deferasirox	3 (18.8)
Decitabine	2 (12.5)
Deferoxamine	2 (12.5)
Azacitidine	2 (12.5)
Granulocyte-colony stimulating factor	1 (6.3)
Prednisone	1 (6.3)
Rituximab	1 (6.3)
Coenzyme Q10	1 (6.3)
Furosemide	1 (6.3)
Previous pharmacological treatment n (%)	
Erythropoietin	10 (62.5)
Lenalidomide	4 (25.0)
Azacitidine	2 (12.5)
Filgrastim	2 (12.5)
Hypomethylating agents	1 (6.3)
Granulocyte-colony stimulating factor	1 (6.3)
Deferasirox	1 (6.3)
Danazol	1 (6.3)

^a^Total n is greater than sample size due to an error in reporting in which one participant was reported to have two types of MDS: RCMD and del(5q)

*IPSS* International Prognostic Scoring System, *MDS* Myelodysplastic syndrome

### Concept elicitation

#### Symptoms

**Fig. 1:**
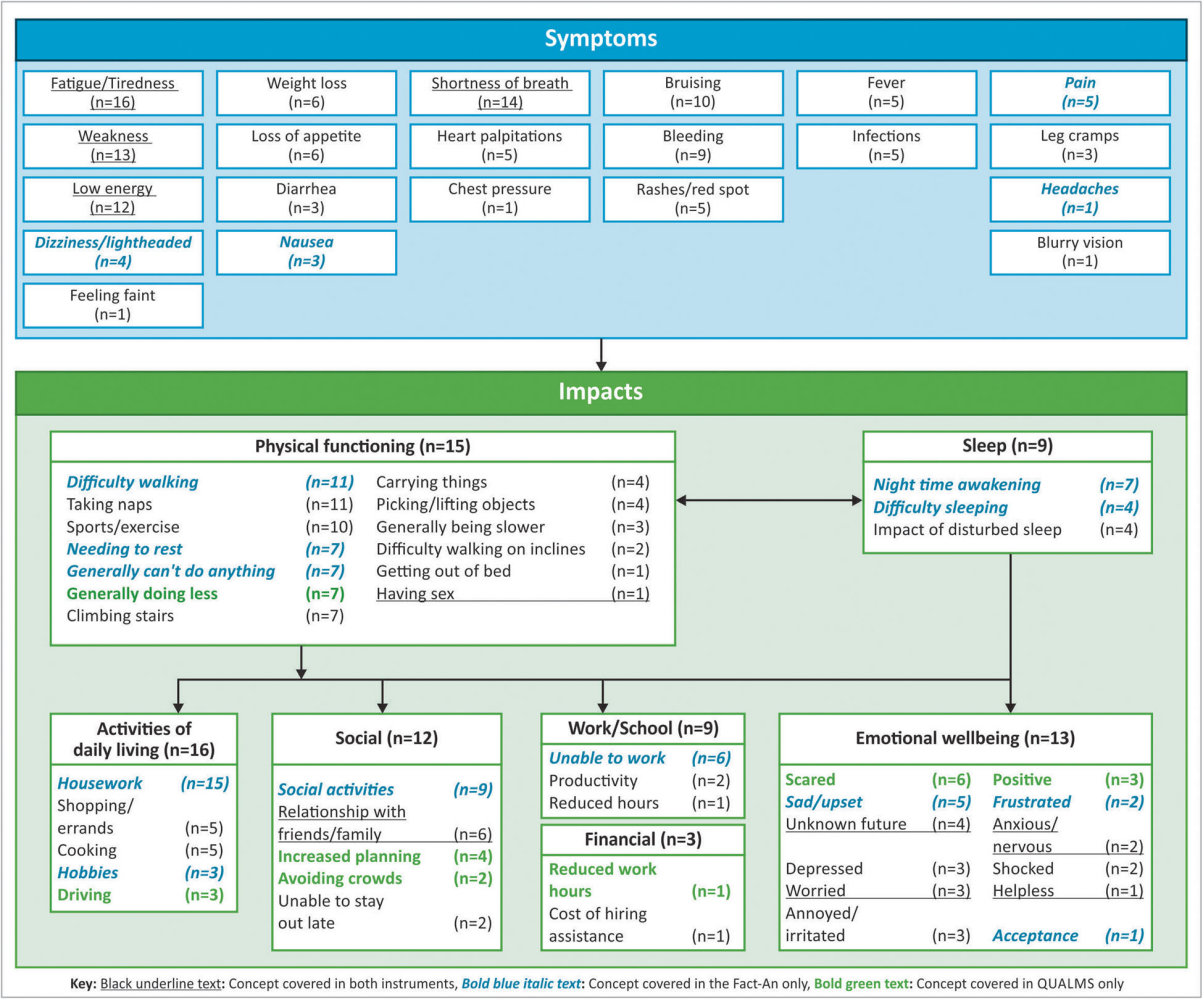
Conceptual model of patients’ experience with MDS

A total of 20 distinct symptoms of MDS were spontaneously reported and two (bruising and weight loss) were reported on probing. The most commonly reported symptoms were fatigue/tiredness (*n* = 16, 100.0%), shortness of breath (*n* = 14, 87.5%), weakness (*n* = 13, 81.2%), and low energy (*n* = 12, 75.0%) (Fig. 1). Not only were these symptoms reported by ≥75% of the participants, but they were considered by participants to be the four most bothersome symptoms of MDS; therefore, the subsequent sections focus on these symptoms. Other key symptoms reported by ≥25% of the sample are presented in Table 2 alongside representative quotes.

**Table 2:** Overview of symptoms emerged during concept elicitation

Symptom	Elicitation	Example quote
Fatigue/Tiredness (*N* = 16, 100.0%)	Spontaneous: 15	*“Oh, just well, I get very tired because my blood count is – I produce no red blood cells. So, I get very tired.”* (63-year old male with INT-1 risk MDS)
	On probing: 1	
Shortness/short of breath (*N* = 14, 87.5%)	Spontaneous: 11	*“Well I don’t think I’ve had a good day. Every day is, uh—I, I’m very tired, very short of breath.”* (61-year old male with INT-1-risk MDS)
	On probing: 3	
Weakness (*N* = 13, 81.3%)	Spontaneous: 2	“*… well my strength level since I’ve been diagnosed with MDS, I’m saying is probably decreased about 35%.”* (84-year old male with low-risk MDS)
	on probing: 11	
Low energy (*N* = 12, 75.0%)	Spontaneous: 12	*“As far as my activity level, I get transfusions. I get two units of blood every two weeks, and when I’m winding down and getting close to needing a transfusion, my energy level gets a lot less.”* (63-year old male with INT-1-risk MDS)
	On probing: Nil	
Bruising (*N* = 10, 62.5%)	Spontaneous: 0	“*… just the slightest touch against a corner or a hard surface and I will end up with a bruise.”* (76-year old female with INT-1-risk MDS)
	On probing: 10	
Bleeding (*N* = 9, 56.3%)	Spontaneous: 3	“*… well sometimes it takes off the top layer of the skin and I will bleed a lot, but the bruise is, um—I mean they’re really not that big and they just, they just go away eventually* (76-year old female with INT-1-risk MDS)
	On probing: 6	
Weight loss (*N* = 6, 37.5%)	Spontaneous: 0	*“I’ve lost, uh, 30 pounds in the, uh—35 pounds in the last six months, uh, due to lack of appetite I guess.”* (71-year old male with INT-1-risk MDS)
	On probing: 6	
Loss of appetite (*N* = 6, 37.5%)	Spontaneous: 4	*“A bad day, uh, I pretty much stay in bed, I’m nauseous, and I can’t eat. And I’m in horrible, horrible pain.”* (64-year old female with INT-1-risk MDS)
	on probing: 2	
Pain (*N* = 5, 31.3%)	Spontaneous: 3	*“But in my bones it’s like in my back and my hip bones, um, my back, my shoulders, um, my legs. It’s muscle and bone pain. Sometimes it feels like somebody is sticking a knife in my bones.”* (64-year old female with INT-1-risk MDS)
	On probing: 2	
Infections (*N* = 5, 31.3%)	Spontaneous: 1	*“I have, um, reoccurring UTIs quite often in the last two or three years.”* (76-year old female with INT-1-risk MDS)
	On probing: 4	
Rashes and red spots (*N* = 5, 31.3%)	Spontaneous: 1	*“no, nothing, ah, unusual other than, you know, I got the impetigo thing which does, you know, affected the skin.” (72-year old* male with INT-1-risk MDS)
	On probing: 4	
Heart palpitations (*N* = 5, 31.3%)	Spontaneous: 5	*“I guess the worse is when I’m lying down in a peaceful rest, I can actually hear it, my heart like pounding, pounding,*
	On probing: Nil	*pounding, pounding, pounding.”* (64-year old female with INT-1-risk MDS)
Fever (*N* = 5, 31.3%)	Spontaneous: 1	*“Sometimes. Um, the doctors, they—um, I think it’s 100.4 if I—it’s lower than a normal person. So if I go above that, then I have to go to the emergency room to bring down my temperature.”* (51-year old male with INT-1-risk MDS)
	On probing: 4	
Dizziness / lightheaded (*n* = 4, 25.0%)	–	“*… dizziness, uh, much more so when, when getting up, when maybe turning quickly.”* (63-year old male with low-risk MDS)

*INT-1 risk* Intermediate-1-risk, *MDS* Myelodysplastic syndrome

##### Fatigue/tiredness

All 16 participants reported fatigue/tiredness as a symptom of MDS of which, 15 participants reported spontaneously and one when probed. Participants used the terms ‘tired’ (*n* = 14, 87.5%) and/or ‘fatigue’ (*n* = 7, 43.7%) interchangeably for this symptom indicating that participants perceived these terms to be the same. Most participants (*n* = 14, 87.5%) generally indicated that their experience of fatigue/tiredness was severe, with some having to stop what they were doing to sit down or sleep. Frequency of fatigue/tiredness was reported by 14 participants, most indicated experiencing fatigue/tiredness everyday/constantly (*n* = 8, 57.1%). Participants highlighted that fatigue/tiredness was closely related to blood transfusions; specifically, eight participants (50%) described how fatigue/tiredness worsens towards the end of transfusion cycle and/or improves following a blood transfusion.

*“ … it really happens on a daily basis at the end of the life of a blood transfusion. After I receive a blood transfusion, there is no fatigue for about a week or two, maybe three*.” [84-year old male with low-risk MDS]

##### Shortness of breath

Fourteen participants reported experiencing shortness of breath as a symptom of MDS, 11 reported this concept spontaneously and three when probed. Participants used the terms ‘short of breath’ (*n* = 9, 64.2%), ‘out of breath’ (*n* = 2, 14.2%), ‘difficult to breathe’ (*n* = 1, 7.1%), ‘can hardly breathe’ (*n* = 1, 7.1%), and having to ‘regain stability in [my] breathing’ after exertion (*n* = 1, 7.1%). Ten participants commented on the frequency of occurrence and the majority of them (*n* = 7, 70.0%) experienced shortness of breath daily. Of the nine participants who commented on duration, most (*n* = 7, 77.8%) described shortness of breath lasting no longer than 10 min, two (22.2%) stated that shortness of breath lasted all day. Nine participants also commented on severity with most (*n* = 7, 77.8%) indicating that it was severe (‘very difficult to breathe’, ‘really hard to breathe’, ‘feel like I’m going to pass out’). Thirteen participants commented on triggers for shortness of breath; climbing stairs (*n* = 8, 61.5%), walking (*n* = 6, 46.2%), doing household chores (*n* = 3, 23.1%), and carrying things (*n* = 3, 23.1%) were most commonly mentioned.

*“I can’t go upstairs. Every time I go upstairs I get out of breath. It’s really hard to breathe. I can’t catch my breath for the most part … ”* [67-year old male with intermediate-1-risk MDS]

##### Weakness

Thirteen participants reported weakness as a symptom of MDS, two (15.3%) reported spontaneously and 11 at probing. Participants used the terms ‘losing muscle strength’ (*n* = 4, 30.7%), and ‘weakness’ or feeling ‘weaker’ (*n* = 2, 15.3%). Five participants commented on severity, two of which (40.0%) indicated it to be severe (‘severe weakness,’ ‘pretty severe’), and three (60.0%) described it as not severe (‘not horrible’). Of the four participants who reported locations of weakness, three (75.0%) mentioned weakness in their legs, two (50.0%) in arms, one (25.0%) in knees, and one (25.0%) in the entire body.

*“I can walk, but I don’t feel like I can. I get weak in my knees … .You feel weak just all over*.” [64-year old female with intermediate-1-risk MDS]

Four participants discussed factors that worsened the weakness, which included coming to the end of their blood transfusion cycle (*n* = 2, 50.0%), physical activity (*n* = 1, 25.0%), and when they did not eat (*n* = 1, 25.0%).

##### Low energy

Twelve participants spontaneously mentioned low energy as a symptom of MDS. Participants used the terms ‘no energy or a lack of energy or low energy levels’ (*n* = 7, 58.3%), ‘lethargic’, ‘less energetic’, ‘not as much stamina’, and/or ‘wiped out’ (*n* = 1, 8.3% for all). Only three participants (25.0%) reported the severity and all described the symptom as severe (‘extreme lack of energy’ or ‘pretty severe’).

*“It’s pretty severe … we have a housekeeper now. I really don’t have the energy to clean my house … two weeks of chemo is really, really low* energy.” [71-year old female with low-risk MDS]

Similar to tiredness/fatigue, participants mentioned that energy levels depleted as they got to the end of their blood transfusion cycle and improved following a blood transfusion (*n* = 5, 41.6%). Six participants (50.0%) described less energy in relation to fatigue/tiredness and two (16.6%) described with weakness; thus, while participants used different descriptors, participants may be using a collection of terms to describe the overall same experience. Unfortunately, this was not explored in any further detail during the interview.

#### Impact

Participants discussed the impact of MDS on the following seven high-level HRQoL domains: activities of daily living (ADL) (*n* = 16, 100.0%), physical functioning (*n* = 15, 93.8%), emotional wellbeing (*n* = 13, 81.3%), social functioning (*n* = 12, 75.0%), sleep disturbance (*n* = 9, 56.3%), impact on work (*n* = 9, 56.3%), and financial impact (*n* = 3, 18.8%) (Fig. 1). The subsequent sections focus on the HRQoL domains reported to be impacted by ≥75% of participants. Table 3 presents example quotes used by participants to describe the impact of MDS on their lives (HRQoL domains reported by ≥25% of the sample only).

**Table 3:** Overview of impacts of MDS on functioning and health-related quality of life

Domain, overall frequency count, n (%)	Elicitation	Example quote
Activities of daily living (ADL, *n* = 16, 100.0%)	Spontaneous: 14	“*… it’s kind of mentally debilitating because I don’t consider myself that old. I’m 51. But, you know, I should be able to do just household chores and stuff around the house, but I can’t do it.”* (51-year old male with INT-1-risk MDS)
	On probing: 2	
Physical Functioning (*n* = 15, 93.7%)	Spontaneous: 15	*“I used to walk my dog and, um, I found that if I even go a half a block with him my heart starts racing.”* (71-year old female with low-risk MDS)
	On probing: 0	
Emotional wellbeing (*n* = 13, 81.2%)	Spontaneous: 7	*“Um, knowing, you know, knowing the unknown, you know, is, is hard to deal with … and speaking with the doctor last week, out of all the 14 cases that have been studied now and treated, nobody survived. So that’s kind of hard to—it doesn’t give you a lot of hope when you’re looking forward.”* (51-year old male with INT-1-risk MDS)
	On probing: 6	
Social functioning (*n* = 12, 75.0%)	Spontaneous: 6	*“I guess I haven’t felt like, uh, you know, uh, doing some things with the family. I’d rather, um, sit at home and, uh, you know, fall asleep in front of the TV.”* (71-year old male with INT-1-risk MDS)
	On probing: 6	
Sleep disturbance (*n* = 9, 56.2%)	Spontaneous: 4	“*What would impact my sleep would be the iron chelator that I take for the haemochromatosis which comes along as the iron overload for the blood transfusion sometimes that causes leg cramps... And it wakes you up in the middle of the night sometimes*.” (63-year old male with INT-1-risk MDS)
	On probing: 5	
Work impacts (*n* = 9, 56.2%)	Spontaneous: 5	*“I retired at 66, but I hadn’t planned on retiring at that point. But it was at the point where there were mornings where I just didn’t want to get out of bed to go to work.”* (68-year old female with low-risk MDS)
	On probing: 4	

*MDS* Myelodysplastic syndrome

##### Activities of daily living

Difficulties with housework/chores (*n* = 15, 93.8%) was the most frequently reported impact on ADLs. Impact on shopping/errands (*n* = 5, 31.3%), cooking (*n* = 5, 31.3%), hobbies (*n* = 4, 25.0%), and driving (*n* = 3, 18.8%) were also mentioned.

*“I stopped cooking altogether a few years ago. And I either pick up things that are easy to put together or I pick up things that I don’t have to do anything with* … ” [68-year old female with low-risk MDS]

The most commonly reported symptoms impacting ADLs were fatigue/tiredness (*n* = 11, 68.8%) and shortness of breath (*n* = 5, 31.3%).

##### Physical functioning

Participants expressed a need to take naps/rest (*n* = 14, 93.3%), had difficulty walking (*n* = 11, 73.3%), difficulty participating in sport and exercise (*n* = 10, 66.7%), difficulty climbing stairs (*n* = 7, 46.7%), generally not being able to do anything (*n* = 7, 46.7%), and generally doing less (*n* = 7, 46.7%) to be the most frequently reported impacts.

*“I was a very active person. I biked. I hiked. I took tai chi. I took yoga. And I can’t do those things anymore*.” [64-year old female with intermediate-1-risk MDS]

Similar to ADLs, commonly reported symptoms impacting physical functioning were fatigue/tiredness (*n* = 11, 73.3%) and shortness of breath (*n* = 10, 66.7%).

##### Emotional wellbeing

The most frequently reported emotional impacts were feeling scared (*n* = 6, 46.2%) and sad/upset (*n* = 5, 38.46%). In contrast, three participants (23.0%) talked about feeling positive as they were not greatly impacted and there was sufficient support/information available (*n* = 3, 23.1%), and one participant (7.7%) mentioned how he had emotionally accepted his MDS.

*“MDS is a terrifying disease, because you have no control in it. … there’s just not many cases of it, so it’s, it’s just not studied that well … I’ve visited two hematologists. They can’t tell you that much about it. It’s just, it’s* terrifying.” [64-year old female with intermediate-1-risk MDS]

##### Social functioning

Participants most frequently talked about the impacts on their ability to participate in social activities (*n* = 12, 75.0%) including inability to attend functions, meetings, a theatre group, or go out for dinner, etc. Four participants (33.33%) cited fatigue/tiredness as the reason for this.

*“ … I was a very full, energetic lady, always running and doing stuff. … always involved in a lot of organizations and doing this and that. But now it’s like, you know, I can’t … I get really tired and I was really* exhausted.” [64-year old female with intermediate-1-risk MDS]

#### Concept saturation

Concept saturation was approached in the total sample with most of the symptoms and impact concepts being spontaneously elicited in the first two sets of interviews. Among symptoms, only fever and headaches emerged in the final set of interviews (one participant each), both of which have been identified as related to MDS in the literature [31, 41, 42]. Given the non-specific nature of these symptoms, it may be possible that others had experienced headaches and/or fever but not attributed it to their MDS. Among impact concepts, only limitations in self-care (*n* = 1, 6.2%), gardening (*n* = 2, 12.5%) and short-term memory (*n* = 1, 6.2%) emerged in the final set of interviews. Similarly, difficulties with housework and chores (inside and outside of the house) and self-care have been described in the MDS literature [43], supporting them as relevant to the MDS patient experience. Impacts to short-term memory, however, were not.

### Cognitive debriefing

#### FACT-An anemia subscale

##### Understanding

The FACT-An items were well understood by majority of the participants. There were some instances of items explicitly not being understood by participants; but in all cases this only applied to one participant. Affected items included: items 30 (‘feelings of listlessness’), 32 (‘trouble starting things’), 33 (‘trouble finishing things’), 44 (‘motivation to do usual activities’), and 46 (‘frustrated by being too tired’) (Supplementary Figure 1a). At the participant-level, no individual participant explicitly misunderstood more than two items. However, these participants seemed to have challenges with the CD process and putting items in their own words, rather than demonstrating a lack of understanding of the items themselves.

##### Relevance

Most of the concepts assessed by FACT-An were relevant to most participants’ experience of MDS, especially items 28 (‘I feel fatigue’), 31 (‘I feel tired’), and 34 (‘I have energy’), which were relevant to all 16 participants. However, items 39 (‘I get headaches’), 41 (‘I have pain in my chest’), and 42 (‘I am too tired to eat’) were reported as not relevant by ≥50% participants (Supplementary Figure 1b). At the participant-level, conceptual relevance was low for three participants, who deemed ≥50% of the items not relevant.

##### Response options

On an item-level, participants generally understood and were able to use the response options without difficulty. After completion of the FACT-An anemia subscale, fifteen participants were asked about the general suitability of the response options and majority (*n* = 9, 60.0%) confirmed the response options were easy to use. The remaining six participants (40.0%) reported having trouble using response options; two (13.3%) due to difficulties choosing between similar response options, one (6.6%) due to relevance of the ‘not at all’ option; other participants did not specify what they found difficult about using the response options.

##### Recall period

All the participants demonstrated understanding of the recall period (‘as it applies to the past 7 days’). Five of nine participants (55.5%) felt the recall period of 7 days was appropriate to capture the most relevant information about MDS and the remaining four (44.4%) mentioned that the past 7 days might not be a reflection of how they felt generally as it depended on where they were in the transfusion cycle. Six participants (37.5%) demonstrated some difficulty adhering to the recall period (e.g. thinking back to a time that month when they had experienced the concept), whereas seven participants (43.7%) said it was easy.

#### Qualms

##### Understanding

Most of the QUALMS items were well understood by majority of the participants. Evidence of misunderstanding was apparent for item 17 (‘sense of gratitude’) and item 37 (‘afraid of treatment failure’), but no more than two participants (12.5%) for each item. At the participant-level, no participants explicitly misunderstood more than two items. However, one participant (6.2%) was unable to verbalize clear understanding for nine items (Supplementary Figure 2a).

##### Relevance

Of 38 QUALMS items, 29 were described as relevant to their MDS experience by ≥50% of participants. Only item 10 (‘medical appointments’) was relevant to all 16 participants. Reported relevance was particularly low for items 34 (‘afraid of losing job’) and 38 (‘too tired to take care of a loved one’), with these items being reported as relevant by one and two participants, respectively. However, this is unsurprising given that none of the participants were currently working and it was unknown whether the participants were responsible for taking care of a loved one. At the participant-level, three participants reported that > 50% of the items were not relevant to their experience of MDS (Supplementary Figure 2b).

##### Response options

On an item-level, participants generally understood and were able to use the response options without difficulty. After completion of the QUALMS, ten participants commented on the response options, with six (60%) reporting the response options as easy to use and four (40%) reporting some difficulty selecting a response option but not providing any further reason as to why.

##### Recall period

Of nine participants who discussed the relevance of the recall period, six (66.7%) commented that due to the variable nature of symptoms, their experiences over the past week did not necessarily reflect their overall experience of MDS [44, 45].

#### Concept mapping

The symptoms and impacts identified during the qualitative CE exercise were mapped on to the QUALMS and FACT-An (in its entirety) questionnaires to assess their conceptual coverage. Collectively the QUALMS and FACT-An provided a sufficient assessment of the most bothersome and most frequently reported symptoms of MDS (tiredness/fatigue, shortness of breath, weakness, and low energy) and assessed each of the HRQoL domains reported to be impacted by participants. Importantly, both instruments together provided sufficient coverage of important impacts to physical functioning (such as, the need to take naps or rest and difficulty walking), although ADLs could be better understood with an assessment of impact to housework/chores (Fig. 1). Moreover, the majority of FACT-An (85%) and QUALMS (76%) items were deemed to be relevant for ≥50% of participants during the cognitive debriefing interviews, further supporting the conceptual relevance of the instruments.

## Discussion

The present study was conducted to identify the symptoms and HRQoL impacts important to patients with lower-risk MDS and assess content validity of the QUALMS and FACT-An anemia subscale in this population. As comprehensive qualitative research/interviews in rare diseases such as MDS is sparse in the published literature, the present research is an important addition to the existing body of evidence.

Amongst concepts emerging from the CE interviews, fatigue/tiredness was the most prominent symptom to patient’s MDS experience as it was reported by all 16 participants and described as the most bothersome symptom by majority of the patients, which is in agreement with the published literature [25, 31, 41, 46]. Moreover, participants in the present study highlighted that severity and frequency of fatigue increased towards the end of their transfusion cycle and improved following transfusion [31, 46].

Shortness of breath was the second most common and bothersome symptom reported by participants, also consistent with the findings of prior qualitative research [25, 43, 47]. Weakness and low energy were also reported to be most bothersome by a few patients. Other symptoms that were reported by patients included pain, bruising, bleeding, infections, fever, and heart palpitations. In line with the literature, participants described a significant impact of these symptoms on ADLs and physical functioning [31, 41, 43, 46, 48, 49]. Most of these impacts were attributed to fatigue/tiredness, shortness of breath, weakness and low energy, further supporting the finding that participants reported these symptoms as most bothersome.

Given the spectrum of symptoms and impacts experienced, these qualitative data also provide an empirical basis for selection of individual concepts for analysis within the larger domains. For example, participants clearly prioritized fatigue and shortness of breath not only as highly frequent, but also as highly relevant and burdensome. Therefore, analysis of these specific items may further elucidate when total- or domain-level findings are driven by these concepts, or when meaningful effects on these concepts could be masked by indifference to other, less-important concepts in the aggregate.

The CD exercise yielded the evidence that both the QUALMS and FACT-An anemia subscale were comprehensive, comprehensible, and patients were easily able to complete the questionnaires. Analysis of concept coverage confirmed that all concepts were relevant to patients with lower-risk MDS to some extent. Although three items (assessing headaches, chest pain and feeling too tired to eat) were deemed as ‘not relevant’ by ≥50% of the sample, they were still considered relevant to over a third of participants and may be relevant to the wider MDS population. Moreover, headache, chest pressure and loss of appetite were spontaneously discussed by participants during the CE exercise, indicating their importance to the MDS experience. Few participants reported that concepts such as emotional wellbeing, thoughts on treatments, thoughts on the future were missing from the FACT-An questionnaire; however, FACT-G includes items assessing emotional concepts, which was not debriefed during the interview due to time constraints. Additionally, conceptual overlap between fatigue and tiredness items was reported, although including multiple items assessing the same underlying concept would serve to promote the reliability of the measure.

While the QUALMS and FACT-An item stems differ, using stems of “how often” and “how much” respectively, their response scales both provide five categorical options. Most participants found them easy to use, and the most commonly articulated issue was in choosing between similar options. Recent systematic reviews of response scales have found that 5-category verbal rating scales (VRS) are generally informative and discriminate well [50], supporting the appropriateness of the response scales for these instruments.

Both the QUALMS and FACT-An had a recall period of 7 days. Although shorter recall periods are typically preferable to minimize recall error and recency effects [51], the appropriateness of a recall period is determined by the phenomena of interest (e.g., variability) and the purpose of the assessment/context of use (in this case as a means of evaluating treatment efficacy/patient response to treatment) [44, 45]. Evidence suggests that the reliability and validity of symptom assessments utilizing a 7-day recall period is often comparable to those using a shorter (24-h) recall period [52]. In the present study, the majority of participants commented on the changing nature of MDS where symptom severity was found to be associated with the transfusion cycle and some suggested that the 7-day recall period may not be best for capturing the true burden of MDS. These findings highlight the importance of the timing of instrument administration relative to treatment and transfusion; that is, given transfusions alleviate the symptoms experienced, administering the instruments just prior to treatment or the transfusion cycle will likely help to capture the true burden of MDS. Considering the timing of transfusion relative to assessments may also improve interpretation of scores. Moreover, a 7-day recall period can be considered more appropriate for capturing the impact on non-daily events or opportunities (e.g., social functioning, recreational activities) [44, 45].

PRO instruments are designed to capture HRQoL data to explain how patients feel or function with their disease and/or treatment. Additionally, HRQoL may be a predictor of clinical outcome [53–56], thus an important parameter of response evaluation [57, 58]*;* therefore, PRO endpoints are becoming increasingly critical in clinical research. Assessment of a PRO instrument’s content validity through patient centric research with qualitative interviews is important for selecting outcome measures that assess concepts that are important to patients. Findings of the present study provide evidence that the QUALMS and FACT-An anemia subscale assess many relevant concepts especially the most frequent and bothersome symptoms among participants. These findings support use of these instruments in a low or intermediate-1-risk MDS population for symptom and functional assessment in clinical research or practice. Though poor HRQoL is frequently observed in patients with lower-risk MDS, it is only partially explained by anemia [41, 53] and assessment of HRQoL in MDS has been recommended by various stakeholders [57–60]. While lower-risk MDS represents a greater proportion of MDS population, robust HRQoL data are limited due to small sample size [61, 62], selection bias [41, 61, 62], and inclusion of patients with higher-risk MDS or AML [18, 25, 53–55, 61, 63]. Therefore, use of a PRO instrument that is comprehensive and comprehensible for patients with lower-risk MDS is necessary.

Evidence from the CE exercise allowed the development of a comprehensive conceptual model that captures symptoms and their impacts on seven HRQoL domains. As expected, concepts elicited during CE quite closely mirrored those included in the QUALMS and FACT-An.

One of the limitations of the present study was a sample that may not be fully generalizable. Although, a quota sampling approach was used to ensure a representative sample of patients with lower-risk MDS, due to rarity of the condition and the challenges experienced in recruitment, not all quotas were met. Therefore, Caucasians, well-educated patients, and those with high-level of functioning (based on ECOG score) were over-represented. Despite this, majority of the patients (*n* = 11, 68.75%) were relapsed/refractory to ESA treatment, which may reflect a population recruited for clinical trials of lower-risk MDS. Another limitation was that the FACT-G sections of the FACT-An were not included in the CD exercise to mitigate participant burden. Findings of the present study should not be extrapolated to the wider MDS population, since the study did not include patients with higher-risk MDS, whose experience may differ due to different disease course and survival than that of patients with lower-risk MDS.

## Conclusion

The present comprehensive qualitative interview study showed that the QUALMS and FACT-An anemia subscale demonstrated strong face and content validity in a lower-risk MDS population. These instruments were very well understood and largely relevant to this population. Overall, the QUALMS and FACT-An are considered appropriate instruments for assessments of symptoms and functioning/HRQoL in patients with lower-risk MDS. Understanding of concepts most important and relevant to patients will help development of novel treatments, which is an unmet need in rare disease management.

## Supplementary information

**Additional file 1: Supplementary Figure 1a**. FACT-An anemia subscale cognitive debriefing results: understanding for all items

**Additional file 2: Supplementary Figure 1b**. FACT-An anemia subscale cognitive debriefing results: relevance for all items

**Additional file 3: Supplementary Figure 2a**. QUALMS Cognitive debriefing results: understanding for all items

**Additional file 4: Supplementary Figure 2b**. QUALMS Cognitive debriefing results: relevance for all items. *Footnote to figure 2b:* Unclear, the participants who either provided a response to the item (which may indicate that they understood the item) but did not expand on their understanding of the concept or wording; or participant who simply repeated the item wording as written. Not asked, not asked due to time constraints

## Data Availability

The data sharing policy of Janssen Pharmaceutical Companies of Johnson & Johnson is available at https://www.janssen.com/clinical-trials/transparency. As noted on this site, requests for access to the study data can be submitted through Yale Open Data Access (YODA) Project site at http://yoda.yale.edu.

## Abbreviations

ADL: Activities of daily living
AML: Acute myeloid leukemia
CD: Cognitive debriefing
CE: Concept elicitation
ECOG: Eastern Cooperative Oncology Group
ESA: Erythropoiesis-stimulating agents
FACT-An: Functional Assessment of Cancer Therapy-Anemia
FACT-G: Functional Assessment of Cancer Therapy-General items
HIPAA: Health Insurance Portability and Accountability Act
HRQoL: Health related quality of life
IPSS: International Prognostic Scoring System
IRB: Independent Review Board
MDS: Myelodysplastic syndromes
PRO: Patient-reported outcomes
QUALMS: Quality of life in myelodysplasia Scale
QUALMS-BF: Quality of life in myelodysplasia -benefit finding
QUALMS-E: Quality of life myelodysplasia-economic burden
QUALMS-P: Quality of life myelodysplasia-physical burden
RBC: Red blood cell
