# Supplementary figures and images for: Content validity of patient-reported outcomes for use in lower-risk myelodysplastic syndromes
Source: J Patient Rep Outcomes. 2020 Aug 26;4:69. doi: 10.1186/s41687-020-00235-4 (PMC7450032; doi:10.1186/s41687-020-00235-4)

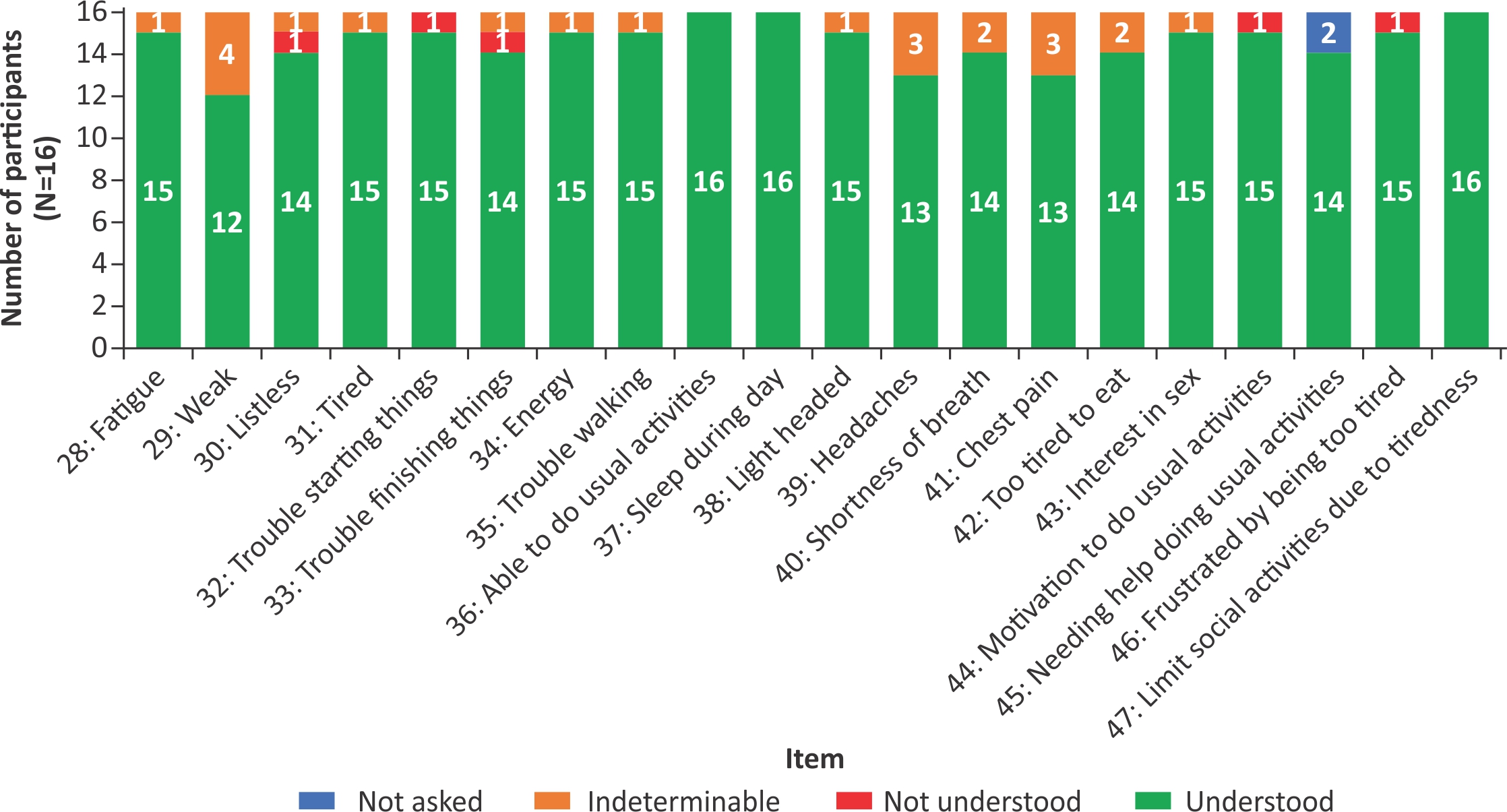

Supplement: Supplementary file 1 — Additional file 1: Supplementary Figure 1a. FACT-An anemia subscale cognitive debriefing results: understanding for all items [file 41687_2020_235_MOESM1_ESM.jpg]

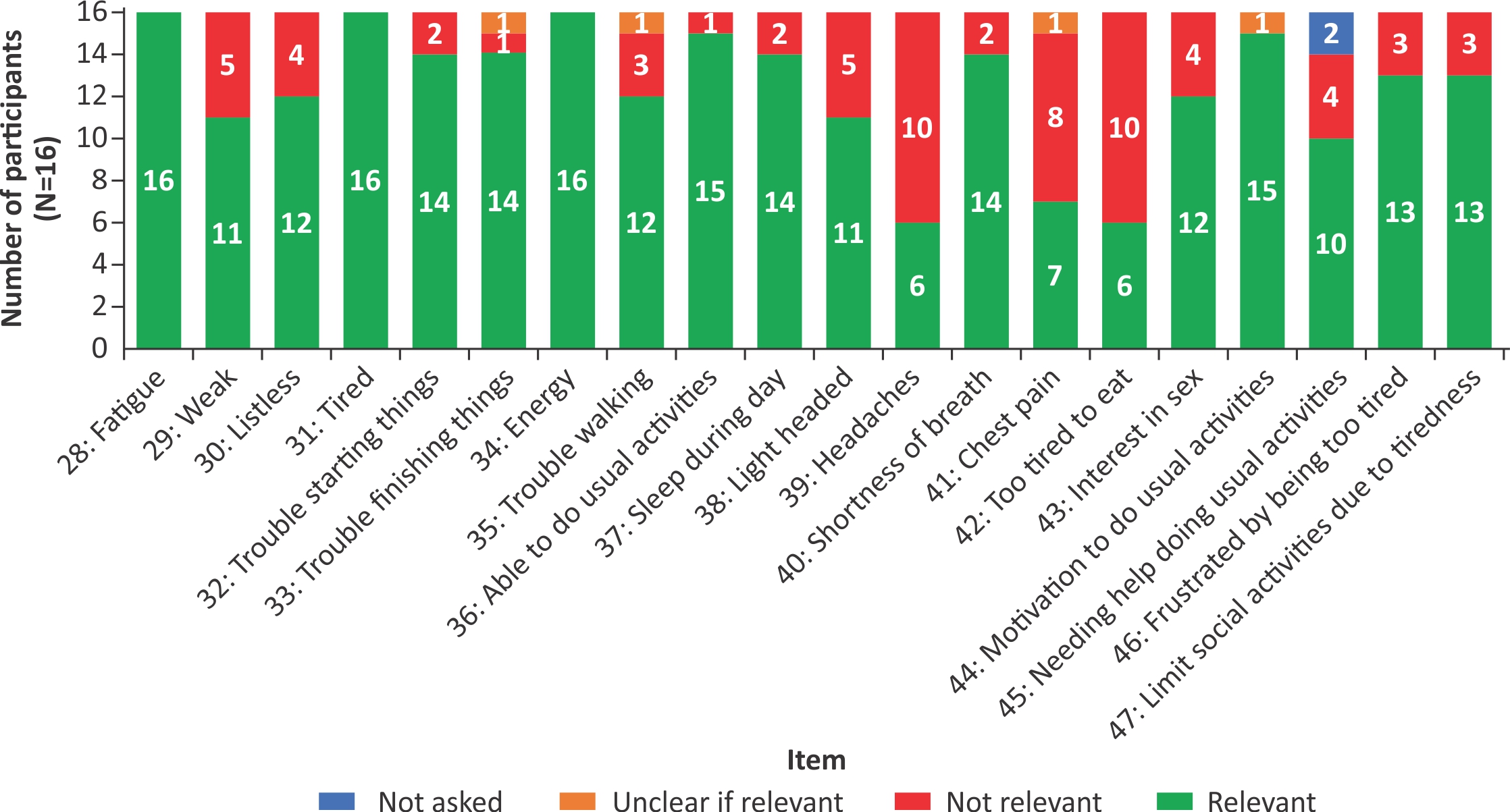

Supplement: Supplementary file 2 — Additional file 2: Supplementary Figure 1b. FACT-An anemia subscale cognitive debriefing results: relevance for all items [file 41687_2020_235_MOESM2_ESM.jpg]

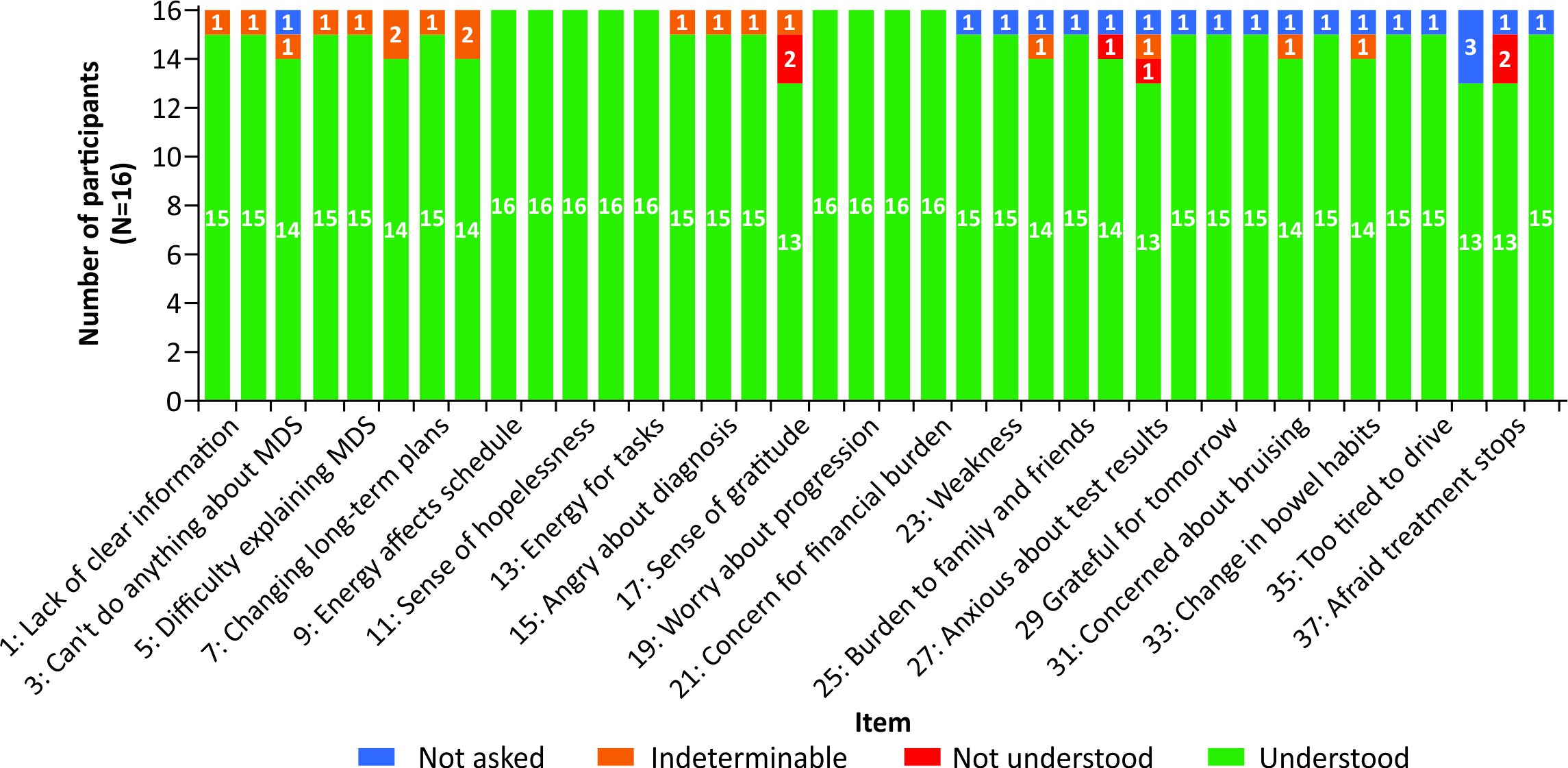

Supplement: Supplementary file 3 — Additional file 3: Supplementary Figure 2a. QUALMS Cognitive debriefing results: understanding for all items [file 41687_2020_235_MOESM3_ESM.jpg]

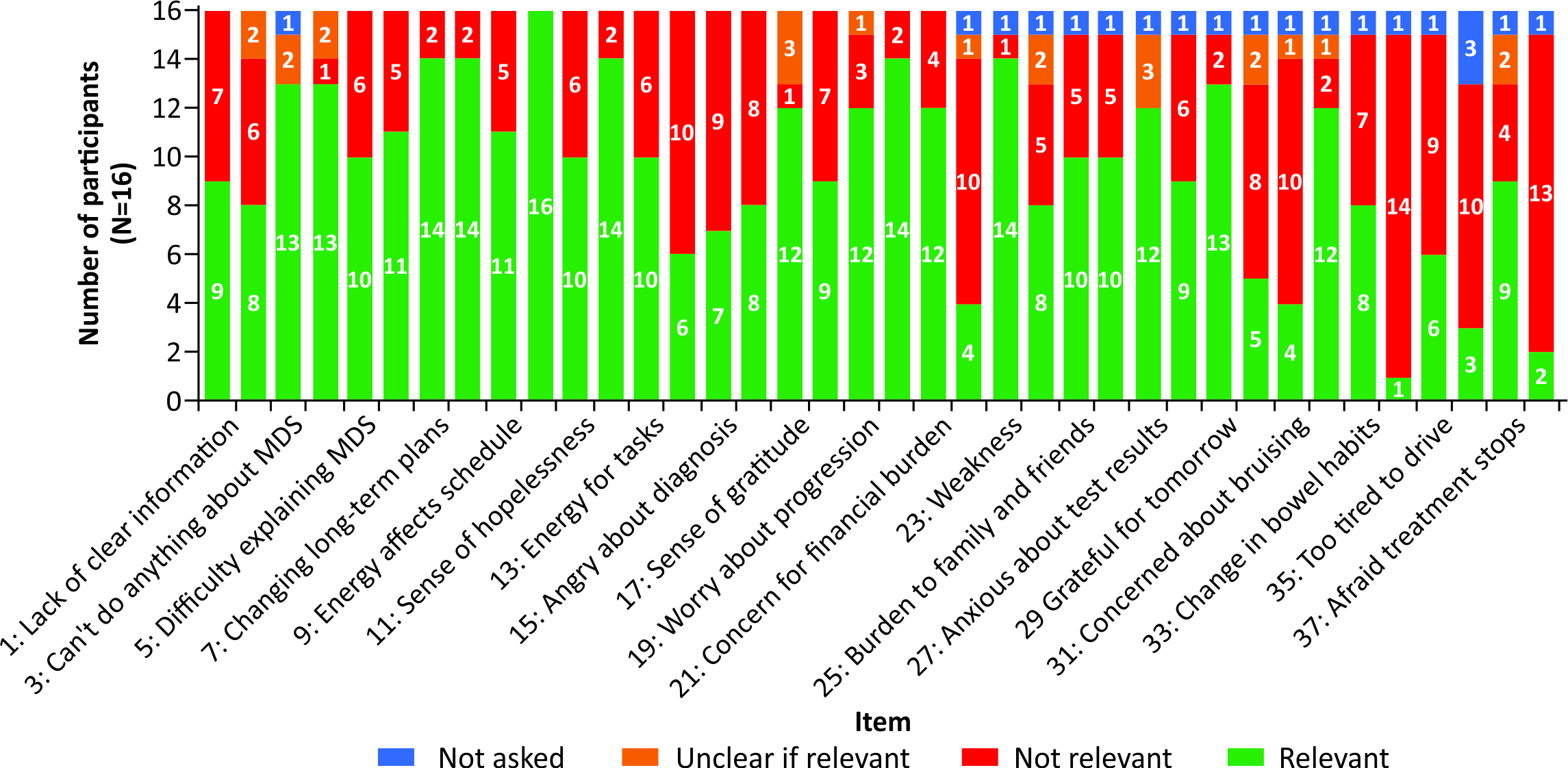

Supplement: Supplementary file 4 — Additional file 4: Supplementary Figure 2b. QUALMS Cognitive debriefing results: relevance for all items. Footnote to figure 2b: Unclear, the participants who either provided a response to the item (which may indicate that they understood the item) but did not expand on their understanding of the concept or wording; or participant who simply repeated the item wording as written. Not asked, not asked due to time constraints [file 41687_2020_235_MOESM4_ESM.jpg]
